# Nicotinism

**Published:** 1877-10

**Authors:** 


### NICOTINISM.

The London *Record* says, M. Mauriac, Surgeon of the Hospital du Middi, has just added another to the special diseases of smokers. He has described, under the title of *plague des fumeurs*, a morbid change of the mucuos membrane of the mouth —a special psoriasis. This lesion may degenerate into epithelioma; and, according to M. Mauriac, cancer of the lips and and tongue has often no other origin than this. Both are common among men, and very rare, as might be supposed, among women.

